# The interplay between satiation and temptation drives cleaner fish *Labroides dimidiatus* foraging behavior and service quality toward client reef fish

**DOI:** 10.1093/beheco/araf131

**Published:** 2025-11-06

**Authors:** Zegni Triki, Xiang-Yi Li Richter, Ana Pinto, Antoine Baud, Sandra A Binning, Mélisande Aellen, Yasmin Emery, Virginie Staubli, Nichola Raihani, Redouan Bshary

**Affiliations:** Institute of Biology, University of Neuchâtel, Emile-Argand 11, Neuchâtel 2000, Switzerland; Institute of Ecology and Evolution, University of Bern, Baltzerstrasse 6, Bern 3012, Switzerland; Department of Biology, University of Konstanz, Universitätsstraße 10, Konstanz 78464, Germany; Institute of Biology, University of Neuchâtel, Emile-Argand 11, Neuchâtel 2000, Switzerland; Institute of Biology, University of Neuchâtel, Emile-Argand 11, Neuchâtel 2000, Switzerland; Département de Sciences Biologiques, Université de Montréal, 1375 Ave. Thérèse- Lavoie-Roux, Montréal, QC H2V 0B3, Canada; Institute of Biology, University of Neuchâtel, Emile-Argand 11, Neuchâtel 2000, Switzerland; Institute of Biology, University of Neuchâtel, Emile-Argand 11, Neuchâtel 2000, Switzerland; Institute of Biology, University of Neuchâtel, Emile-Argand 11, Neuchâtel 2000, Switzerland; Department of Experimental Psychology, UCL, 26 Bedford Way, London WC1H 0AP, United Kingdom; School of Psychology, University of Auckland, 23 Symonds St, Auckland 1010, New Zealand; Institute of Biology, University of Neuchâtel, Emile-Argand 11, Neuchâtel 2000, Switzerland

**Keywords:** cheating, cooperation, decision-making, game theory, mutualism, wild fish

## Abstract

Supply and demand affect the values of goods exchanged in cooperative trades where high demand typically leads to a higher cost. An exception has been described in the marine cleaning mutualism involving the cleaner fish *Labroides dimidiatus* and its variety of “client” coral reef fishes. Cleaner fish feed on clients' ectoparasites (ie gnathiid isopods) but prefer eating clients' mucus instead, which constitutes cheating. Here, we provide field observations, followed by a set of laboratory experiments with real client fish and Plexiglas feeding plates as surrogates for clients. In the field and in three experiments with real clients, we found that satiated cleaner fish were more cooperative, even though low hunger levels should make them less dependent on cleaning interactions. Similarly, the more abstract version of the cleaner–client experiments using Plexiglas plates offering two food types as stand-ins for client ectoparasites and mucus showed that satiation led cleaner fish to feed more against their preferences—an indicator of cooperative behaviour. However, this outcome occurred only if the temptation to eat the preferred food was low. When temptation to cheat was high, cleaner fish did so. We provide a further general support to these findings with a game-theoretic model. Many mutualisms involve food as a commodity. Thus, identifying foraging decision rules will enhance our understanding of how individuals adjust to variations in market conditions in real-time rather than playing a fixed strategy based on average market conditions.

## Teaser

Our study revealed intriguing behaviours in cleaner fish, highlighting how satiation and temptation influence their cleaning interactions. Cleaner fish generally prefer to “cheat” and eat the client's mucus rather than clean off parasites. We show that as cleaner fish get hungrier and the temptation gets stronger, they cheat more often. Understanding these behaviours can provide insights into how animals adjust their foraging strategies in response to changing environments.

## Introduction

The values of goods or services exchanged in human economic markets follow the rules of supply and demand (Smith 1974). Typically, goods become more expensive when they are in high demand. Similar economic rules of supply and demand used to predict payoff distributions among cooperating humans also apply to other species within the framework of biological market theory and its emphasis on partner choice (Noë et al. 1991; Noë and Hammerstein 1995). Evolutionary models of biological market theory invariably predict that as demand goes up, so does the cost (Noë and Hammerstein 1994; Schwartz and Hoeksema 1998; Johnstone and Bshary 2008; De Mazancourt and Schwartz 2010; Akçay et al. 2012; Grman et al. 2012). The models assume that individuals evolve to play optimal strategies when market conditions are generally stable across generations (Noë and Hammerstein 1994). In contrast, empirical studies manipulate current conditions to test how individuals flexibly adjust to changes in a market. For example, lycaenid butterfly larvae produce more attractive volatiles and offer more sugary food secretions to incite more ants to tend and protect them if current ant numbers are low (Leimar and Axén 1993; Axén et al. 1996). Similarly, vervet monkeys give more grooming to a group member who has been experimentally made the sole provider of high-quality food than if a second group member can also provide access (Fruteau et al. 2009). Various reviews on partner choice in cooperative interactions summarize the available evidence that individuals adjust their behaviour to current market conditions (Jones et al. 2012; Barclay 2013; Hammerstein and Noë 2016).

A notable exception to the market law of supply and demand has been described in the marine cleaning mutualism involving the cleaner fish *Labroides dimidiatus* and its client fishes (Triki et al. 2022). There are two main types of client fish: visitors and residents. Visitor clients are typically large-bodied species with access to multiple cleaning stations, allowing them to choose among different cleaner fish. In contrast, resident clients are usually smaller, territorial species that rely exclusively on their local cleaning station and have no choice of alternative partners (Bshary 2001). Clients offer a good—ectoparasites—to the cleaner fish, which, in turn, provide a service to clients by removing their ectoparasites. These iterated interactions yield a net benefit to clients (Ros et al. 2011; Waldie et al. 2011; Demairé et al. 2020). This benefit is derived in part due to clients making cleaner fish eat against preference: despite gnathiids being the most abundant ectoparasites of clients in the Indo-Pacific and the most found in the stomachs of cleaner fish (Grutter 1997), the latter prefer eating the clients' mucus that protects scales and skin over gnathiid ectoparasites (Grutter and Bshary 2003). We refer to the observation that cleaners prefer mucus over gnathiids as the “temptation” to cheat. Beyond any preference, eating mucus has the advantage of covering the entire surface of the client, while ectoparasites need to be searched for on the client's body. Consequently, cleaner fish scrape indiscriminately on clients if the latter are anesthetized (Bshary and Grutter 2002c). Non-predatory clients can reduce the likelihood that cleaner fish eat their mucus by either punishing a cheating cleaner by chasing them or swimming off and seeking a different cleaner fish for their next cleaning interaction. This behaviour encourages cleaner fish to act more cooperatively in future interactions (Bshary and Grutter 2002b, 2005). The ratio of ectoparasite-to-mucus consumption by cleaner fish is thus a key variable describing their service quality (Bshary and Noë 2023). As mucus consumption often causes the occurrence of client “jolts’ (ie, an abrupt whole-body movement or convulsion) in response to cleaner fish mouth contact (Bshary and Grutter 2002b), jolt frequency is an easily observable correlate of cleaner fish service quality.

Based on biological market theory, cleaner fish are expected to cheat less frequently when demand for cleaning services is low, as losing a client is detrimental to them. This is anticipated to result in a lower jolt rate. By contrast, when demand is high and many clients compete for cleaning services, cleaner fish can afford to cheat more often. This is expected to result in an increased jolt rate. The ratio of cleaner fish to clients can cause shifts in client fish demand for cleaning services. This ratio may naturally vary due to demographic changes (mortality and recruitment) or environmental perturbations (Triki et al. 2018), and can also differ between habitats (Wismer et al. 2014; Triki et al. 2019). A game-theoretic model developed to analyze cleaner-client interactions in the biological market context revealed that the link between supply-to-demand ratios and client jolt rates is complex in this cleaning mutualism system (Triki et al. 2022). Instead, the outcome depends on how the marginal benefits of mucus consumption over ectoparasite consumption change with increasing food abundance, in conjunction with how readily clients elevate their tolerance levels to mucus consumption based on their ectoparasite load. For instance, in laboratory experiments, we see that clients are more tolerant of cleaner fish eating mucus when the ectoparasite load is high than when it is low (Bshary and Grutter 2002c). That is, if the marginal benefits decline more quickly, more slowly, or at the same rate as the corresponding rise in client tolerance, then the client jolt rates will be lower, higher, or remain unchanged, accordingly (Triki et al. 2022).

The biological market model put forward by Triki et al. (2022) is not explicit about the mechanisms underlying cleaner fish's decision to cheat (eat mucus) or cooperate (eat ectoparasites) during cleaning interactions. However, there is an obvious candidate mechanism that links variation in food abundance to cleaner fish adjustment of service quality: satiation levels. If cleaner fish are rare, each individual cleaner fish faces a high demand for cleaning services from clients. In the study by Triki et al. (2022), cleaner-client ratio was explicitly manipulated by reducing the population of cleaner fish to 50%, resulting in an increase of the demand for cleaning services without affecting client jolt rates (Triki et al. 2022). However, the remaining 50% of cleaner fish did not change the frequency and duration of their cleaning interactions in response, which means that clients had, on average, fewer cleaning interactions than expected. On the physiological level, client fish from cleaning stations where cleaner fish were removed recorded lower hematocrit levels, an indicator of anemia likely caused by hematophagous ectoparasites such as gnathiids (Demairé et al. 2020). This suggests that clients harbored more ectoparasites, allowing cleaner fish to consume them at higher rates, thereby achieving greater levels of satiation. Importantly, this scenario lacks straightforward empirical validation for two assumptions: an increased ectoparasite load in clients and the heightened feeding rate of cleaner fish. However, it does predict that cleaner fish adjust their service quality, as indicated by clients' jolt rates, according to their own satiation levels. If that is the case, cleaner fish should modify their service quality in response to fluctuations in their satiation levels, whether these arise from stochastic short-term changes in client visitation rates or longer-term changes in cleaner-to-client ratios.

To develop predictions about how satiation levels affect service quality in the short term, we can consider the long-term predictions from the biological market model proposed by Triki et al. (2022). The main difference between short-term and long-term variation in cleaner satiation levels is that the latter is caused by changes in cleaner-to-client ratios, while the former results from a combination of service quality and random fluctuations in client visitation at the cleaning station. For example, several large-bodied clients harboring many ectoparasites (Grutter 1994) seeking cleaning services in short succession will cause the interacting cleaner fish to be temporarily more satiated than average. In this state, the cleaner fish is less immediately dependent on cleaning interactions with clients and may, therefore, be less willing to clean for the next few interactions. This shift could lead to an increase in the cleaner fish's mucus feeding rates, ie, cheating rates. Conversely, as cleaner fish become more satiated, both the benefits of foraging and the marginal benefits of consuming mucus over ectoparasites also decrease. Most importantly, this reduction in the temptation to eat mucus is not offset by clients being less likely to respond aggressively or with fleeing. Instead, clients will respond to cleaner mucus feeding based on probabilities that correspond to current overall market conditions rather than the cleaner’s momentary satiation level. As long as clients continue to punish or switch cleaner fish partners, the model's logic converges with optimal foraging theory and its focus on trade-offs between foraging and risk (Milinski and Heller 1978; Cuthill and Houston 1997): satiated cleaner fish should become risk-averse and hence reduce their cheating rates.

Here, we investigated how stochastic variation in client visit rates and the resulting short-term fluctuations in cleaner fish satiation levels affected their service quality in four research parts. In the first part, we used field observation data to test whether interacting first with large client fish, a correlate for temporarily increased food intake and thus increased satiation, influenced cleaner fish service quality in subsequent cleaning interactions. Cleaner service quality was measured by recording the client's jolt rate (Bshary and Grutter 2002c) in subsequent interactions. In the second part, we conducted a series of laboratory experiments in which we exposed both satiated and hungry cleaner fish to real client fish and quantified the client jolt rates to measure cleaner fish service quality. In the third part, in another set of laboratory experiments, we utilized Plexiglas plates with food as substitutes for real client fish. In this setup, we varied the satiation levels of the cleaner fish and presented them with Plexiglas plates offering fish flake mixture items and prawn items as stand-ins for gnathiid ectoparasites and mucus, respectively. Each plate was attached to a lever, allowing the experimenter to remove it as soon as a cleaner fish consumed a preferred prawn item, thus imitating a client fish swimming away after a cleaner fish ate its mucus. The experiment measured the cleaner fish's willingness to feed against their preference and eat more flakes, which corresponds to eating ectoparasites rather than mucus in nature. This experimental system has been successfully applied in the past and effectively captures key features of real cleaner-client cleaning interactions (Bshary and Grutter 2005, 2006; Pinto et al. 2011; Salwiczek et al. 2012; Gingins et al. 2013). We could therefore evaluate how the baseline willingness to feed against preference affected adjustments in food choices when satiated. In the final and fourth part, we developed a game-theoretical model as a complementary approach to explore how the ratio of eating non-preferred versus preferred food is influenced by the interaction between variations in the temptation to cheat and satiation levels. The model builds on the one published by Triki et al. (2022). Here, we explore how changes in the benefits of cheating as a function of satiation levels may lead to either an increase or a decrease in cheating rates when the cleaner fish is satiated.

## Methods

### Part 1: Field observations

Field observations on natural cleaner-client interactions collected from May to July 1998 and 1999 at Ras Mohammed National Park, in Egypt (see Bshary et al. 2011), were reanalyzed here for current purposes. Bshary et al. followed and observed 16 individual adult cleaner fish (adults are identifiable by a bold black stripe from the mouth through the eye to the tail, contrasting with bright blue dorsal and ventral regions; Randall et al. 1997) on the reef for 3 to 4 h per cleaner fish, during which they recorded all instances of cleaner-client fish cleaning interactions. Cleaners spend about 20% of their time interacting with clients (Barbu et al. 2011), and hardly ever ignore visitor clients. Such visitors are not always available, and their appearance is subject to random variation. For every cleaner–client interaction, Bshary et al. (2011) recorded the client fish species, the duration of the interaction, and the number of client fish body jolts. Bshary et al. transcribed the field observation data into sequences of cleaner–client interactions. This enabled us to reconstruct interaction sequences with large visitor clients, which correlate with a temporary increase in cleaning demand due to random fluctuations. Additionally, large clients (high-quality clients) are more profitable as ectoparasite load correlates with body size (Grutter 1994, 1995), these strings of interactions naturally lead to longer interaction durations and higher feeding rates by the cleaner fish (Grutter 1995). We defined a “high feeding rate sequence” as one in which there were at least three consecutive visits from visitors, and cleaning occurred for a minimum total duration of 60 s. We first identified all of the high feeding sequences from these field observations, and then manually extracted all situations when the following interaction occurred with a non-predatory resident client. Strings of interactions and follow-up interactions that included (resident) predatory clients were deliberately excluded, as cleaner fish tend to improve their service quality and reduce their mucus-feeding frequency towards predator clients (see Bshary et al. 2011). Thus, including such interactions could have biased the data.

The aim was to test whether cleaner fish satiation level affected service quality, inferred from client jolt rates, where a jolt indicates poor service because the cleaner cheated by feeding on mucus instead of removing parasites, whereas lower jolt rates reflect higher service quality. To do so, we attributed the status “satiated” to cleaner fish interacting with residents immediately after having a high feeding sequence, and the status “hungry” to cleaner fish interacting with residents without such a recent high feeding sequence. To simplify the analysis, we averaged the observation values for jolt rate per client species and the cleaner fish status. This yielded a matched design with 17 resident client species, where we had a value for a given species when interacting with satiated cleaner fish and when interacting with hungry cleaner fish.

### Part 2: Interactions with real client fish (Laboratory Experiments 1 to 3)

We conducted laboratory-based experiments in 2010, 2017 and 2018 at the Lizard Island Research Station, Great Barrier Reef, Australia (see summary Table 1). Using barrier nets and hand nets, we collected female adult cleaner fish (*L. dimidiatus*)—identified as the second-largest individuals in the size-based social hierarchy, ranking just below the dominant male (Robertson 1972)—and three client species: staghorn damsels (*Amblyglyphidodon curacao*) as a representative of a small territorial resident, and monocle bream (*Scolopsis bilineatus*) and striated surgeonfish (*Ctenochaetus striatus*) as a representative of visitors with still relatively small home ranges from the surrounding reefs of Lizard Island and transported them to the lab facilities on the island. In captivity, we housed fishes in individual aquaria (minimum size: 69 × 25 × 30 cm) with PVC tubes of various sizes as shelters. All aquaria had continuous flow of water and air filters. We fed the cleaner fish daily with mashed prawn spread on Plexiglas plates that served as surrogates for client fish. Client fishes each had their own diet: we fed the *A. curacao* daily with commercial fish flakes, *S. bilineatus* with diced prawn, and *C. striatus* with a mixture of flakes and prawn smeared on Plexiglas plates. At the end of the lab experiments, we released all caught fishes back at their respective sites of capture.

**Table 1.** Overview of Experiments 1 to 7 on cleaner fish.

Partner	Experiment	Year	Species tested	Sample size (N)	Duration & trials	Treatments
**Real client fish**	1	2010	16 *L. dimidiatus* + 16 *A. curacao*, 16 *S. bilineatus*, 16 *C. striatus*	16 cleaner fish, each paired with 3 different client spp.	2 sessions/day × 10 min each, for 6 consecutive days	Hungry vs. satiated, client species counterbalanced
	2	2017	20 *L. dimidiatus* + 20 *C. striatus*	20 pairs	2 sessions per fish × 15 min each	Hungry vs. satiated,
	3	2018	20 *L. dimidiatus* + 20 *C. striatus*	20 pairs	2 sessions per fish × 15 min each	Hungry vs. satiated,
**Plexiglas plates as surrogates for client fish**	4	2004	16 *L. dimidiatus* (Plexiglas plates)	16 cleaners	14 trials over 2 days (7 trials/treatment/day)	Hungry vs. satiated
	5	2017	18 *L. dimidiatus* (Plexiglas plates)	18 cleaners	20 trials over 2 days (10 trials/treatment/day)	Hungry vs. satiated
	6	2018	18 *L. dimidiatus* (Plexiglas plates)	18 cleaners	16 trials over 2 days	Hungry vs. satiated
	7	2018	20 *L. dimidiatus* (Plexiglas plates)	20 cleaners	40 trials over 4 days (10 trials/treatment/flake mix per day)	Hungry vs. satiated × two flake concentrations (10% vs. 40%)

All data collected in these experiments has not previously been published.

All fish we used in these experiments were adults, selecting based on coloration and body size (see Randall et al. 1997). In all experiments, a cleaner fish and a client fish could interact with each other for ten minutes in Experiment 1 and for 15 min in Experiment 2. We video-recorded these interactions. In the “satiation” condition, cleaner fish were allowed to feed on food plates five minutes prior to interacting with clients; whereas in the “hungry” treatment, cleaner fish had been fed last the previous afternoon prior to the interaction with the client. Every cleaner fish experienced both “satiation” and “hungry” treatments, while we ensured a counterbalanced design to control for potential treatment order bias. Researchers who analyzed the videos were blinded to the condition of the cleaner fish. In each video, we measured the total duration(s) of the cleaning interactions and the number of client fish body jolts in contact with a cleaner fish's mouth (Bshary 2001).

#### Experiment 1 (2010)

Experiment 1 was conducted in July 2010 and involved 16 cleaner fish, 16 *A. curacao*, 16 *S. bilineatus*, and 16 *C. striatus*. Each cleaner fish was paired with three different client reef fishes (one individual of each species). For each treatment (hungry or satiated), we tested every cleaner fish twice a day (morning and afternoon sessions) with the same client fish partner, with each testing session lasting 10 min (ie, 20 min in total per day). Experiments were therefore conducted over six consecutive days. We counterbalanced the order of which species a cleaner fish interacted with as well as the treatment order among our 16 cleaner fish test subjects to account for any sequence effects in experience and client ectoparasite loads.

#### Experiment 2 (2017)

Experiment 2 was conducted in July 2017. We tested 20 cleaner fish with 20 *C. striatus*. Each cleaner fish had its own *C. striatus* partner. In Experiment 2, cleaner fish interacted twice with their client partner (once under the hungry treatment and once under the satiated treatment), with each session lasting 15 min. The session length was extended by five minutes compared to Experiment 1 (2010), as the earlier data showed that interactions with satiated cleaners tended to be short. By increasing observation time, we aimed to collect more behavioral data and reduce variance in jolt frequency estimates. Treatment order was counter-balanced as for Experiment 1.

#### Experiment 3 (2018)

Experiment 3 was conducted in July 2018. We tested 20 cleaner fish with 20 *C. striatus*. The methods followed the same procedure as in Experiment 2.

### Part 3: Interactions with Plexiglas plates as surrogates for real client fish (laboratory Experiments 4 to 7)

The aim of the experiments with Plexiglas plates was to test adult cleaner fish for their foraging decisions when hungry vs satiated. In contrast to interactions with clients, where jolts are a correlate of cleaner fish eating mucus (Bshary and Grutter 2002a), the experimenters can see for each foraging decision precisely what the cleaner chose to eat. The plates offered prawn items and flake items. We know from previous studies that cleaner fish prefer prawn over flakes (Bshary and Grutter 2005). Therefore, eating a less preferred flake item is a proxy for choosing to eat an ectoparasite, which constitutes cooperating. In contrast, eating a preferred prawn item is a proxy for choosing to eat client fish mucus, which constitutes cheating.

Following methods by Bshary and Grutter (2005), we trained cleaner fish subjects that foraging against their preferences increases food intake. To do so, we trained them with Plexiglas plates offering 12 flake items and two prawn items. Cleaner fish could freely forage on flake items, but we withdrew the plate upon the consumption of a prawn item. The plate was returned within a minute, and the cleaner could forage again until it ate the second prawn item, leading to the removal of the plate until the next trial. In total, we ran six training trials over 2 days. Over the course of six trials, all cleaner fish ate several flake items before eating a prawn item and hence experienced the diverging consequences of food type choices. From the study by Bshary and Grutter (2005), six trials were sufficient for cleaner fish to show significant learned feeding against preference.

In total, we conducted the experiment four times: Experiment 4 in June 2004, Experiment 5 in July 2017, Experiments 6 and 7 between February and July 2018 (see summary Table 1). In all of these experiments, tests and manipulations occurred during daytime hours between 8:00 and 17:00. Test trials invariably consisted of presenting a plate with three flake items and three prawn items on it, where the cleaner eating a prawn item led to the immediate removal of the plate. The plates were 12 × 7 cm in size, except for experiment 6 (where they were 9 × 9 cm). At the end of the trials, we calculated separately the feeding against the preference ratio for the two treatments by dividing the sum of flake items by the sum of prawn items consumed during the trials. Another similarity between the four experiments was that cleaner fish experienced testing days where they could eat extra flake items before each trial to create the “satiated” treatment, and testing days without extra food to create the “hungry” treatment. The order of treatments was counterbalanced across cleaner fish within each experiment. Intertrial intervals were about 30 min.

In Experiment 4, we tested 16 adult cleaner fish. We had 14 test trials over 2 days with seven trials per treatment and day. In Experiment 5, we tested 18 adult cleaner fish. We ran 20 test trials over 2 days, with ten trials per treatment and day. In Experiment 6, we tested 18 adult cleaner fish, and we performed a total of 16 test trials per fish over 2 days. Lastly, in Experiment 7, we tested 20 adult cleaner fish. In contrast to Experiments 4 to 6, we manipulated the concentration of flakes in the flake-prawn mixture. Previously, a rough estimation was that we prepared this mixture as one-third volume of fish flakes mixed with two-thirds volume of prawn. Furthermore, the flake brands made available through the research station changed between years (with no tracking of brand names), and it appeared from the results of the first three experiments that this may have affected the cleaner fish's baseline willingness to feed against preference. Previous research has shown that flake concentration affects feeding against preference, with cleaner fish less willing to eat flake mixture items containing high flake concentration (Gingins et al. 2013). This baseline willingness, in turn, may affect how cleaner fish adjust their feeding against preference as a function of satiation levels. Therefore, in Experiment 7, we tested whether the flake content in the flake mixture affected cleaner fish foraging decisions when hungry compared to when satiated. We weighed prawn and flakes to the nearest mg to produce two precise flake-prawn mixtures. One mixture contained 10% flake and 90% prawn, while the other consisted of 40% flake and 60% prawn. We tested the fish over 4 days with 10 test trials per fish and per day. Each fish faced ten trials per satiation level treatment (hungry vs satiated) and flake content in the flake mixture (10 vs 40%). We counterbalanced the treatment order among the tested fish.

### Statistical analyses

We used the open-source software R Version 3.6.3 (R Core Team 2022) to run the statistical analyses and generate the figures. Given that we had multiple experiments that were run in different periods and by different researchers, we opted for analyzing every dataset generated from these experiments separately.

#### Part 1

For field observations, we used a linear mixed effect model, R package: LMER, (Kuznetsova et al. 2017), with the mean jolt rates (number of jolts per 1 s of cleaner–client interaction) of 17 different client species as the response variable, cleaner satiation state (hungry vs satiated) as a fixed factor, and client species as a random factor.

#### Part 2

For Experiments 1 to 3 (with client fish), we analyzed client fish jolt rate as the number of jolts per 1 s of interaction as the response variable. For Experiment 1, the statistical model had satiation treatment (hungry vs satiated), client species (*A. curacao*, *S. bilineatus*, *C. striatus*) and their interaction term as fixed factors. In Experiment 1, we had cleaner-client pair identity and the test session (morning or afternoon) as random factors. For Experiments 2 and 3, we included satiation treatment as a fixed factor and cleaner-client pair identity as random factor. In Experiments 1 and 3, we fitted Generalized Linear Mixed Models using Template Model Builder (glmmTMB in R language; Brooks et al. 2017) for beta distribution (values between 0 and 1) due to zero-inflated data. In contrast, we fitted an LMER model for data from Experiment 2.

#### Part 3

For Experiments 4 to 7 (Plexiglas plates), we also fitted LMER models with flake to prawn ratio as the response variable, satiation treatment (hungry vs satiated) as a fixed factor, and cleaner identity as a random factor. Additionally, in Experiment 7, we had another factor which was the fish flake concentration in the flake mixture. Therefore, we had flake content (10 vs 40%) as another fixed factor in the model for this dataset, as well as the interaction term between flake content and satiation treatment.

For post hoc analyses, we ran emmeans functions in R language (Lenth and Lenth 2016). All model assumptions were met. In some cases, we included data transformation, such as square root (for field data, part1) or log-transformation (for Plexiglas plate experiments, part3), to meet normality and homogeneity of variance assumptions. We provide a detailed step-by-step R code along with the data for further information.

#### Part 4: Game-theoretic model

We extended the existing theoretical model described in Triki et al. (2022) to help explain the effect of the quality of different food types on cleaner fish service quality as a function of satiation levels. The previous model explored how cleaner fish should adjust service quality (the ratio between ectoparasite removal acts and mucus feeding acts) when an increased demand for cleaning by clients causes an increase in the cleaner's overall access to food and hence a monotonal increase in satiation levels. The model showed that the adjustment depends on how clients adjust their probability to punish a cleaner for a cheating act as a function of increased ectoparasite load. In general, increased ectoparasite load should make clients more tolerant (ie more likely to remain even after cleaners eat mucus), but the exact function may vary between species for various reasons (eg differences in maneuverability, Roche et al. 2021, or punishment being co-opted by intraspecific aggressiveness, Soares et al. 2019). Consequently, the model predicts that satiated cleaner fish will behave more cooperatively towards less tolerant client species and more exploitative towards more tolerant client species (Triki et al. 2022). Here, we apply this logic to analyze how short-term variation in satiation may affect the cleaner fish's willingness to feed against preference as a function of the temptation to cheat. The level of temptation is how much cleaner fish prefers a client species' mucus over ectoparasites, or in the plate experiments, it is how much cleaner fish prefers the prawn items over the flake items.

The model description is formulated based on the plate experiments. The cheating rate (*r*) is determined by balancing the benefit of gaining nutrition through eating a prawn item and the cost imposed by the experimenter by withdrawing the Plexiglas plate.


(1) $$\dot{r}=B(x)-C(r,x),$$

where the benefit function B(x) is an S-shaped decreasing function of food intake *x*. The shape of the benefit function is controlled by a parameter γ, which represents the quality of the food.


(2) $$B(x)=1-e^{-10e^{-\gamma x}}$$

Food quality refers to the nutritional value or energetic payoff of each food item. A higher γ value reflects better food quality, such that the higher the γ, the faster the benefits of cheating decline as food intake accumulates.

The cost function takes the form


(3) $$C(r,x)=r(1-e^{-10e^{-10x}}),$$

which is also an S-shaped function that decreases with the cumulated food intake of the cleaner fish *x*, as the marginal benefit of cheating decreases with the level of satiation, and it is proportional to the cheating rate *r*, because the experimenter withdraws the Plexiglas plate every time the cleaner eats a prawn item. The equilibrium cheating rate $r^{*}$ can be calculated by setting $\dot{r}=0$.

## Results

### Part 1: field observations

**Fig. 1.**
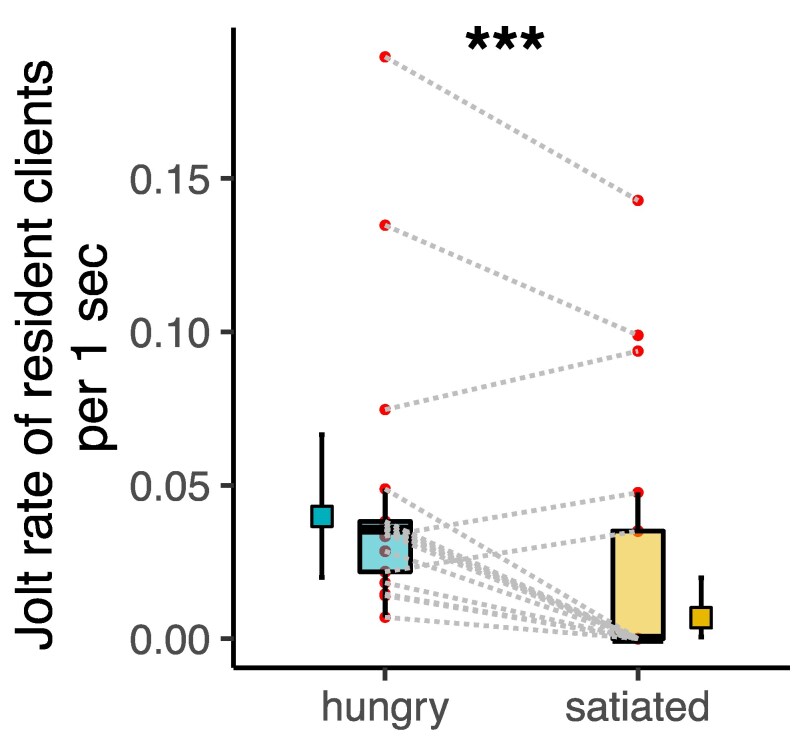
Jolt rates of 17 resident client species during natural cleaning interactions with hungry and satiated cleaner fish. The plot shows estimated means and 95% CI of model marginal effects next to boxplots of median and interquartile of raw data, and the actual data points. Dashed lines connect data points of the same client species. *** LMER; *P* < 0.001.

Resident clients jolted less frequently if the cleaner fish had previously experienced a high feeding sequence of cleaning interactions with visitor clients (LMER: cleaner state (hungry vs satiated): χ^2^ = 29.84, estimate [low, high 95% Confidence Interval] = −0.117 [−0.16, −0.07], N = 34, *P* < 0.0001, marginal-R^2^ = 0.20, conditional-R^2^ = 0.77, Fig. 1).

### Part 2: Interactions with real client fish (Experiments 1 to 3)

**Fig. 2.**
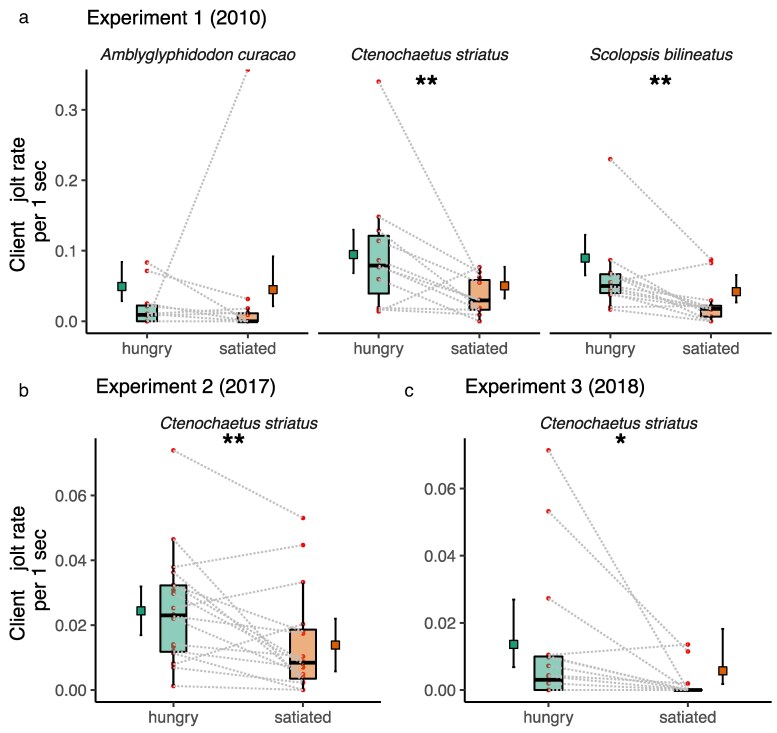
Client fish body jolt rates during cleaning interactions with cleaner fish in laboratory settings (Experiments 1 to 3). a) to c) show estimated means and 95% CI of models marginal effects next to boxplots of median and interquartile of raw data, and the actual data points. Dashed lines connect data points from the same cleaner-client pair (client species: staghorn damsels *A. curacao*, monocle bream *S. bilineatus*, and striated surgeonfish *C. striatus*). a) Pairwise emmeans posthoc test ** *P* < 0.01; b) **LMER; *P* < 0.01; c) * GlmmTMB; *P* < 0.05.

In all three experiments, we found a statistically significant main effect of satiation treatment on client fish body jolt rate, where client fish jolted less frequently when interacting with satiated cleaner fish compared to when interacting with hungry cleaner fish (glmmTMB and LMER: *P* < 0.05, see detailed statistics in Table 2, Fig. 2). In Experiment 1, where we had three different client species tested with cleaner fish, the posthoc test indicated that the effect was more evident in *C. striatus* (emmeans estimate = 0.679, t-ratio = 2.728, *P* = 0.007) and *S. bilineatus* (emmeans estimate = 0.815, t-ratio = 3.199, *P* = 0.002) but not in *A. curacao* (emmeans estimate = 0.097, t-ratio = 0.218, *P* = 0.828) (Fig. 2a).

**Table 2.** Summary table of the statistical outcomes from Experiments 1 to 3 with real client fish.

Fitted model	*N*	Model/distribution	*Chi square (X^2^)*	*P-*value	Marginal-R^2^/Conditional-R^2^
Experiment 1 (year 2010) model syntax (jolt rate ∼ satiation treatment*client species *+* *(1|cleaner identity)* *+* *(1|test session)))*					
Satiation treatment	16	GlmmTMB/beta	**13.887**	**<0.001**	**0.18/0.36**
Client species	16	GlmmTMB/beta	3.983	0.136	**0.18/0.36**
Satiation treatment × Client species	16	GlmmTMB/beta	2.002	0.367	**0.18/0.36**
*Experiment 2 (year 2017) Model syntax (jolt rate ∼ satiation treatment* *+* *(1|cleaner identity))*					
Satiation treatment	20	LMER/Gaussian	**8.313**	**0.004**	**0.10**/**0.62**
*Experiment 3 (year 2018) Model syntax (jolt rate ∼ satiation treatment* *+* *(1|cleaner identity))*					
Satiation treatment	19	GlmmTMB/beta	**3.890**	**0.049**	**0.17**/**0.85**

Indicated in bold are statistically significant *P*-values (alpha ≤ 0.05).

### Part 3: Interactions with Plexiglas plates as surrogates for client fish (Experiments 4 to 7)

**Fig. 3.**
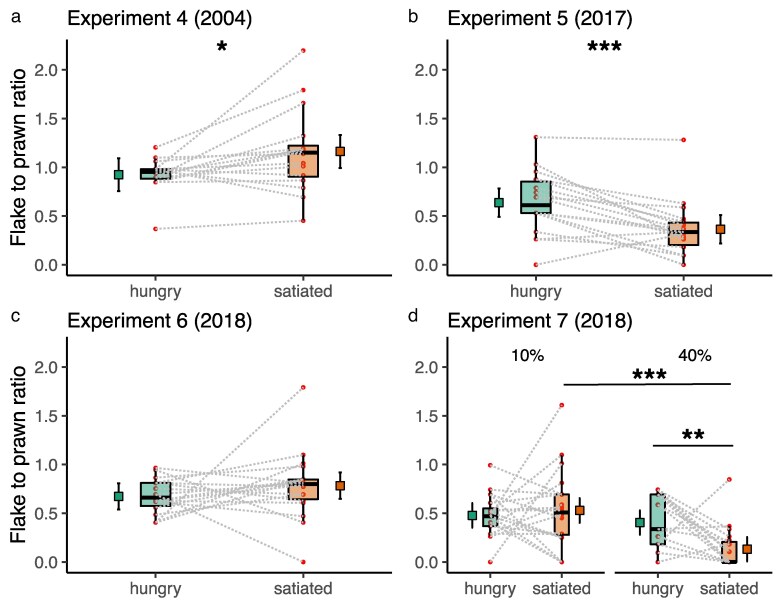
Cleaner fish feeding against preferences as a function of satiation state in four experiments with Plexiglas plates (Experiments 4 to 7). a) to d) show estimated means and 95% CI of models marginal effects next to boxplots of median and interquartile of raw data, and the actual data points. Dashed lines connect data points from the same cleaner fish. d) Experiment 7 had a two-by-two factorial experimental design with two levels of satiation state (hungry vs satiated) and two treatments of flake content (10% vs 40%). a) *LMER; *P* < 0.05; b) *** LMER; *P* < 0.001; d) Pairwise emmeans posthoc test ** *P* < 0.01, *** *P* < 0.001.

Experiments 4 to 7 did not reveal consistent effects of cleaner fish satiation state on their rates of feeding against preference, where the feeding-against-preference ratio reflects the number of less-preferred flake items consumed before the fish “cheat” by eating a highly preferred prawn item (see detailed statistics in Table 3, Fig. 3). For instance, in Experiment 4, cleaner fish fed significantly more against their preferences when satiated than when they were hungry (LMER: *P* < 0.05, Fig. 3a), which agrees with the previous results with real clients where the jolt rates (proxy for cheating) decreases when satiated. However, we had the opposite effects in Experiment 5, where we had less eating against preference when satiated (LMER: *P* < 0.001, Fig. 3b), and no apparent effect of satiation treatment in Experiment 6 (LMER: *P* > 0.05, Fig. 3c).

**Table 3.** Summary table of the statistical outcomes from laboratory experiments with plexiglas plates as surrogates for client fish.

Fitted model	*N*	*Chi square (χ^2^)*	*P-*value	Marginal-R^2^/Conditional-R^2^
*Experiment 4 (year 2004) model syntax (flake to prawn ratio ∼ satiation treatment* *+* *(1|cleaner identity))*				
Satiation treatment	16	**6.445**	**0.011**	**0.12**/**0.43**
*Experiment 5 (year 2017) Model syntax (flake to prawn ratio ∼ satiation treatment* *+* *(1|cleaner identity))*				
Satiation treatment	18	**20.812**	**<0.001**	**0.17**/**0.71**
*Experiment 6 (year 2018) Model syntax (flake to prawn ratio ∼ satiation treatment* *+* *(1|cleaner identity))*				
Satiation treatment	18	1.397	0.237	0.04/0.04
Experiment 7 (year 2018) Model syntax (flake to prawn ratio ∼ satiation treatment*flake content *+* *(1|cleaner identity))*				
Satiation treatment	20	3.412	0.065	**0.23**/**0.30**
Flake content	**20**	**15.356**	**<0.001**	**0.23/0.30**
Satiation treatment × Flake content	**20**	**7.282**	**0.007**	**0.23/0.30**

Indicated in bold are statistically significant *P*-values (alpha ≤ 0.05). All statistical models were LMER.

When we manipulated the mixture's flake content in Experiment 7, we found that cleaner fish presented with the 40% concentrated flakes (high temptation treatment) scored lower flake-prawn ratios than when presented with the 10% flake mixture (low temptation treatment; LMER: *P* < 0.001, Fig. 3d). This effect was driven by a significant interaction between flake content and satiation treatment (LMER: *P* < 0.01, Fig. 3d): under the 40% flake content condition, cleaner fish ate more significantly against their preferences when hungry than satiated (emmeans: estimate = 0.273, t-ratio = 3.214, *P* = 0.002) whereas in the 10% flake content condition, there were no significant differences in feeding against preference between hungry vs satiated cleaner fish (emmeans: estimate = −0.051, t-ratio = − 0.602, *P* = 0.549).

### Part 4: game theoretical model

Our game-theoretic model can explain the results of the experiments with Plexiglas plates. In the model, the equilibrium cheating rate can be calculated with a closed-form expression


(4) $$r^{*}=\frac{1-e^{-10e^{-\gamma x}}}{1-e^{-10e^{-10x}}}.$$

**Fig. 4.**
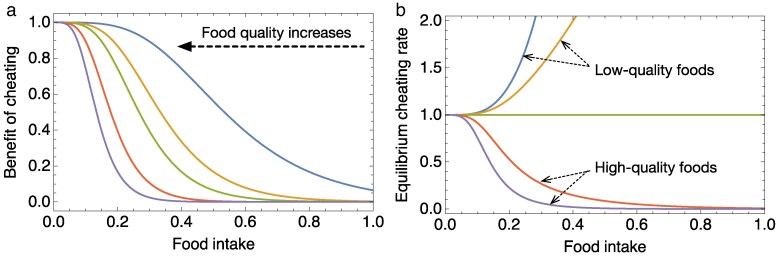
Game theoretical model predictions. a) An illustration of the benefit function, where the benefit of cheating decreases with the quantity of food intake. Lines of different colors correspond to different food quality represented by different *γ* values, which were set to 5 (representing poorest quality), 8, 10, 15, and 20 (representing highest quality) from right to left. b) The equilibrium cheating rate can either decrease or increase with food intake, depending on the quality of the alternative food, represented by the parameter *γ* in the benefit function. The values of *γ* for the blue, orange, green, red, and purple curves are 5, 8, 10, 15, and 20, respectively.

Varying the parameter *γ* in the benefit function changes how fast the benefit of cheating decreases with food intake (Fig. 4a). When the decline of cheating benefit is very slow (corresponding to the low quality of alternative food compared to the preferred prawn), the equilibrium cheating rate increases as food intake increases (Fig. 4b, blue and orange lines), mirroring the results of the second part of Experiment 7 with cleaner fish being less cooperative when satiated than when hungry when the temptation to eat a preferred food was high. In contrast, when the benefit of cheating declines rapidly (corresponding to better quality of the alternative food), the equilibrium cheating rate decreases as food intake increases (Fig. 4b, red and purple lines). These simulation results are similar to the results in Experiments 4 and 7 with cleaner fish eating more often against their preference when satiated than when hungry when the flake concentration in the alternative food was low.

## Discussion

We asked whether satiation affects service quality in cleaner fish, under the assumption that satiation levels may provide a proximate mechanism for why adjustments in levels of cooperation occur in response to short-term changes in biological market conditions. Both field data and lab experiments involving real clients generally show that clients jolt less when cleaner fish are satiated (ie, cleaner fish cheat less when not hungry). The exception to this trend occurred when clients were damselfish. This species jolted less frequently than other species in our experiment (see Fig. 2a). The experiments involving plates and the game-theoretic model added another dimension to the results on real interactions, showing that satiation may have both positive and negative effects on cooperation levels, depending on the cleaner fish's temptation to cheat. These results provide key insights into how animals may use basic proximate mechanisms to flexibly adjust to short-term and long-term changes in the supply-to-demand ratio, potentially in ways that defy human market law.

### Interactions with real client fish

Short-term variations in satiation levels occur largely due to stochastic fluctuations in local demand for cleaning, without any changes in cleaner-to-client ratios. This stochastic variation arises from independent decisions made by individual clients regarding when to visit a particular cleaning station. After a short-term increase in visitation rates, cleaner fish become temporarily more satiated. The results indicate that being satiated leads to cleaner fish causing fewer jolts per time unit. Consequently, they become more cooperative rather than increasing rates of mucus feeding. This result may be predicted when we apply optimal foraging theory (Cuthill and Houston 1997)—and the logic of variable investment in repeated games (Johnstone and Bshary 2008)—to our study system. As long as the marginal benefits of eating mucus are low when cleaner fish are in a satiated state and high when they are in a non-satiated state, satiation should lead to relatively higher levels of cooperation. Indeed, despite the variation in client body size and home range, the effect of satiation on client jolt rate was quite consistent in our experiments. Importantly, when we consider short-term fluctuations in cleaner satiation levels, increased cooperation when satiated enhances payoffs in the near future because good service positively impacts the next interaction with the same client 10 to 40 min later (Bshary and Würth 2001; Soares et al. 2013), ie, when the cleaner fish's satiation level is most likely back to average, considering that their foraging is spread over 2,000 interactions per day (Grutter 1995). In other words, cleaner fish are more cooperative when satiated, which can be viewed as functionally investing in relationships with their clients since such behaviour yields future benefits.

Given that satiation levels affect the cleaner fish's level of cooperation in the short term, it seems likely they also influence the cleaner fish's adjustments to long-term changes in cleaner-to-client ratios. With satiation as a mechanism, an increase in client demand for cleaning services is predicted to cause cleaner fish to provide better service. Conversely, a decrease in client demand is predicted to lead cleaner fish to lower their service quality. Satiation effects would, therefore, oppose market effects. In contrast, client responsiveness to being cheated, another major factor affecting cleaner cooperation (Bshary and Grutter 2002b; Roche et al. 2021), is expected to change according to market effects: clients should be more tolerant when demand for cleaning is high, and less tolerant when demand for cleaning is low (Triki et al. 2022). The relative effect sizes of these two opposing forces will determine how changes in the supply-to-demand ratio will affect cleaner fish cooperation (Triki et al. 2022).

### Plexiglas plate experiments (as surrogates for client fish) and a game-theoretical model

In contrast to the data involving real clients, we found evidence that the willingness of cleaner fish to eat against their preference in the Plexiglas plate experiments both increased and decreased based on satiation levels. Experiment 7 provided crucial insights into the mechanism behind such variable results. When the preference for prawn versus flakes is weak, satiated cleaner fish become more willing to eat flakes against their preference. Conversely, when the preference for prawn is strong due to high flake concentration and/or a particularly distasteful flake mixture for cleaner fish, satiated cleaner fish become less willing to eat against their preference. The game-theoretical model supports this interpretation. In interactions with actual clients, the rationale is inverted, as the less preferred food (ectoparasites) tends to exhibit similar taste characteristics across diverse species, whereas the quality of the preferred food (mucus) is recognized to fluctuate between species (Arnal et al. 2001; Roche et al. 2021), and cleaner fish do show preferences (Grutter and Bshary 2004). Both the empirical results and the model pave the way for new experiments involving actual clients. The hypothesis to be tested is that cleaner fish adjust their service quality according to their satiation level and the ecological properties of client species. Specifically, client species that present a high temptation to consume mucus, such as those with abundant high-quality mucus and few ectoparasites, are expected to jolt more frequently when cleaners are satiated, reflecting reduced service quality. In contrast, client species with lower temptation, with limited lower-quality mucus and higher ectoparasite loads, should jolt less frequently under the same conditions. Future theoretical and empirical extensions could further test how these effects interact with client size, used as a proxy for food patch size, and the presence or absence of bystanders, providing a broader understanding of how satiation and temptation jointly shape service quality.

### Satiation and feeding against preference

Another intriguing research avenue is to adopt a comparative approach to examine whether the motivational mechanisms of cleaner fish have evolved specifically as adaptations to the ecological challenges inherent in cleaning interactions (Kamil and Mauldin 1988; Shettleworth 1993). The challenge of consuming food contrary to one's preferences in order to increase personal food intake in the presence of a preferred option is likely rare in nature. Consequently, we predict that other non-cleaning species would experience difficulty in consuming food against their preferences initially, and this difficulty would be exacerbated when satiated. A well-documented, related phenomenon, observed in humans and other taxa, is referred to as sensory-specific satiety, also known as the “dessert effect.” This phenomenon entails a decrease in pleasure associated with the continuous consumption of the same food or flavor, as compared to that of an unconsumed food or flavor (Rolls et al. 1981; Havermans et al. 2009; Ostojić et al. 2013). Keeping the analogy of the dessert effect, satiated animals should tend to focus on their preferred food. In the case of cleaner fish, this would mean eating mucus or prawn rather than gnathiids or flakes (Grutter and Bshary 2003; Bshary and Grutter 2005). We predict that a reversal of this tendency will enable cleaner fish to functionally invest in future relationships with clients when their needs are met. The evolution of such a motivational mechanism, which adjusts to variations in supply-to-demand ratios over both short and long terms, contrasts with the presumed manner in which humans adapt to market conditions. Indeed, humans possess cognitive abilities that allow them to monitor developments in the market and plan for the future—a capacity that is typically absent in non-human animals, as well as in plants and microbes (Tulving 2005; Raby et al. 2007).

### Conclusions and outlook

Our findings emphasize the significance of examining the processes involved in decision-making to improve our understanding of biological markets. It is essential to investigate the impact of satiation levels across various mutualisms, given that food is a frequently exchanged commodity (Bronstein 1994; Pierce et al. 2002; Bronstein et al. 2006; Kiers et al. 2011). Cleaning mutualism is just one form of so-called protection mutualism, where one class of partner species trades protection for food with the other class of partner species (Bronstein 1994). In mutualisms of protection, a high demand for protection positively influences food availability for the protectors (Axén et al. 1996). As a consequence, protectors may use their food intake rates and resulting satiation levels as a basis for flexible adjustment of service quality. The current study and the study by Triki et al. (2022) demonstrate that cleaner fish do not exhibit behaviour consistent with the predictions of the market law of supply and demand. One would anticipate that a low demand for protection would result in hungry protectors, thereby leading to an improved quality of service compared to that provided by satiated protectors. Consequently, the pertinent question arises: do other protector species exhibit analogous patterns, or do they modify their protective services as anticipated by biological market theory? A potentially distinctive attribute of the *L. dimidiatus* cleaning mutualism is the conflict of interest between the protector and the food provider regarding the protector's dietary choices. This conflict may influence the quality of service in ways that diverge from the expectations established by market law (Bshary and Noë 2023). The effects of other ecological settings on the quality of protection have not been thoroughly investigated; however, they may produce intriguing dynamics. For instance, ant species could theoretically adapt to an increased demand for protection by contributing additional food resources to the colony, thereby promoting more rapid colony growth. This scenario suggests that the advantages for ants form a linear relationship with food availability, as opposed to an asymptotic relationship characterized by diminishing returns when an individual forages for its own sustenance. The manner in which this linear benefit function influences the level of protection offered by ants, as well as the potential role of individual satiation in decision-making processes, presents a compelling avenue for future research.

## Data Availability

Analyses reported in this article can be reproduced using the data and code provided by Triki et al. (2025).
